# Fast GPU-based Monte Carlo code for SPECT/CT reconstructions generates improved ^177^Lu images

**DOI:** 10.1186/s40658-017-0201-8

**Published:** 2018-01-04

**Authors:** T. Rydén, J. Heydorn Lagerlöf, J. Hemmingsson, I. Marin, J. Svensson, M. Båth, P. Gjertsson, P. Bernhardt

**Affiliations:** 1 Department of Radiation Physics, Institute of Clinical Sciences, Sahlgrenska Academy at University of Gothenburg, SE-413 45 Gothenburg, Sweden; 2 Department of Medical Physics and Biomedical Engineering, Sahlgrenska University Hospital, SE-413 45 Gothenburg, Sweden; 3 Department of Oncology, Institute of Clinical Sciences, Sahlgrenska Academy at University of Gothenburg, SE-413 45 Gothenburg, Sweden; 4 Department of Clinical Physiology, Sahlgrenska University Hospital, SE-413 45 Gothenburg, Sweden

**Keywords:** Monte Carlo, GPU, SPECT, OSEM

## Abstract

**Background:**

Full Monte Carlo (MC)-based SPECT reconstructions have a strong potential for correcting for image degrading factors, but the reconstruction times are long. The objective of this study was to develop a highly parallel Monte Carlo code for fast, ordered subset expectation maximum (OSEM) reconstructions of SPECT/CT images. The MC code was written in the Compute Unified Device Architecture language for a computer with four graphics processing units (GPUs) (GeForce GTX Titan X, Nvidia, USA). This enabled simulations of parallel photon emissions from the voxels matrix (128^3^ or 256^3^). Each computed tomography (CT) number was converted to attenuation coefficients for photo absorption, coherent scattering, and incoherent scattering. For photon scattering, the deflection angle was determined by the differential scattering cross sections. An angular response function was developed and used to model the accepted angles for photon interaction with the crystal, and a detector scattering kernel was used for modeling the photon scattering in the detector. Predefined energy and spatial resolution kernels for the crystal were used. The MC code was implemented in the OSEM reconstruction of clinical and phantom ^177^Lu SPECT/CT images. The Jaszczak image quality phantom was used to evaluate the performance of the MC reconstruction in comparison with attenuated corrected (AC) OSEM reconstructions and attenuated corrected OSEM reconstructions with resolution recovery corrections (RRC).

**Result:**

The performance of the MC code was 3200 million photons/s. The required number of photons emitted per voxel to obtain a sufficiently low noise level in the simulated image was 200 for a 128^3^ voxel matrix. With this number of emitted photons/voxel, the MC-based OSEM reconstruction with ten subsets was performed within 20 s/iteration. The images converged after around six iterations. Therefore, the reconstruction time was around 3 min. The activity recovery for the spheres in the Jaszczak phantom was clearly improved with MC-based OSEM reconstruction, e.g., the activity recovery was 88% for the largest sphere, while it was 66% for AC-OSEM and 79% for RRC-OSEM.

**Conclusion:**

The GPU-based MC code generated an MC-based SPECT/CT reconstruction within a few minutes, and reconstructed patient images of ^177^Lu-DOTATATE treatments revealed clearly improved resolution and contrast.

## Background

The Monte Carlo (MC) method provides a unique opportunity for simulating radiation transport. It has been used for many years to mimic medical imaging systems such as gamma cameras [1–3]. However, pure photon transport from the emission site to the final photon absorption in the gamma camera crystal requires substantial simulation times to generate images with low noise. To address this, effective variance reduction techniques have been developed and implemented into MC codes. These time-optimized codes have been successfully used to investigate various aspects of gamma camera performance [1, 4–6].

The faster simulation times achieved with variance reduction techniques also support the use of the MC method in the reconstruction of tomographic images from planar images, i.e., single-photon emission computed tomography (SPECT). Novel studies have shown the potential to increase the image quality of SPECT by using MC-based reconstruction [7–9]. However, the simulation times in MC-based SPECT reconstruction are lengthy due to the need to simulate all forward projections and because the codes are generally written for the central processing unit (CPU), which limits effective parallelization. For a SPECT reconstruction of a 128^3^ patient matrix and 120 projections, a rough estimate is that about 128^3^*120*(100 photons/voxel) have to be simulated per iteration to obtain forward projections with an acceptable image noise, i.e., 25*10^9^ photon emissions per iteration. Despite the use of multiple kernels for parallelization of the code, the simulation times for SPECT reconstruction with CPU-based codes are still long. Nevertheless, using graphics processing units (GPUs) for optimized parallelization might substantially reduce SPECT reconstruction times. Recently, Garcia et al. [10] demonstrated that GPU programming for parallelization of the photon transport in the general GEANT4 code could reduce simulation times by a factor of 70. With this approach, the simulation times in MC-based SPECT reconstruction with GEANT4 were reduced to hours in phantom studies.

In line with the progression of various techniques to speed up the simulation times in Monte Carlo-based SPECT reconstruction, we intended to investigate possible simulation times when using established variance reduction techniques and the Compute Unified Device Architecture (CUDA) language to control the tremendous parallel computing capacity of contemporary Nvidia GPUs. We conducted simulations and measurements for ^177^Lu, which is used in therapeutic applications, such as ^177^Lu-labeled somatostatin analogs or ligands for prostate-specific membrane antigen [11–13]. Due to its emission of photons, the doses absorbed by tumors and critical organs, mainly the kidney and bone marrow, can be estimated from gamma camera measurements [14–16]. However, no clear correlation between absorbed dose and tissue response has been established. One reason for this might be the use of mean absorbed doses for non-homogenous activity distributions [17, 18]. A non-homogenous dose distribution and its effect on the biological response might be possible to account for by the equivalent uniform dose (EUD) approach [19]. However, increased resolution and contrast of the gamma camera are required to substantially improve the accuracy of the dose volume histograms required for EUD. The triple energy window (TEW) methodology is an established approach for correcting for the scattering influence in the images, and the use of distance-dependent point kernels can be used for resolution recovery corrections [20]. The drawback with TEW is that voxel values can be negative and with resolution recovery corrections Gibbs artifacts, i.e., ringing artifacts are introduced in the image. The ringing artifacts are an effect of the suppression of high-frequency components, necessary for the reconstruction not to result in excessive high-frequency noise in the image. Both affect the quantitative accuracy, especially for low uptake volumes such as the bone marrow cavities [20, 21].

Recently, Hippeläinen et al. performed a ^177^Lu SPECT dosimetry study and introduced a more advanced MC-based scattering correction than TEW and demonstrated increased concentration recovery coefficient for attenuated and resolution recovery reconstructed images [22]. However, the authors address that MC-based scatter correction is time-consuming, and therefore, they applied an accelerated version. Consequently, the execution times for MC-based SPECT reconstructions are important to consider, and running the MC codes on GPUs can generate improvements, as demonstrated by Garcia et al. [10].

In this study, we will develop a GPU-based MC code for SPECT reconstructions and compare it with attenuated non-scattered corrected OSEM reconstruction and with reconstruction with resolution recovery correction in phantom studies and in clinical ^177^Lu images.

## Methods

The MC code SARec (Sahlgrenska Academy reconstruction code) was specifically designed for SPECT reconstructions and was written in the CUDA language for computers with graphics processing units (GeForce GTX Titan X, Nvidia, USA). SARec can be used with an arbitrary number of GPUs; in this study, we used four GPUs, which is about the maximum number that can be installed in an ordinary desktop without requiring a special power supply or cooling arrangement. The GPUs are run in parallel for generating the MC-simulated forward projections.

### Photon interactions

In SARec, the photon emission from each voxel in the matrix and its interactions was simulated in parallel. The emission and tracking of the emitted photon to the detection in the crystal were simulated by validated methodologies [1, 4–6, 23, 24]. In SARec, the scattering order for the emitted photons, i.e., the number of interaction points, can be arbitrarily chosen. For ^177^Lu, minimal gain in image quality is obtained for scattering orders higher than three (data not shown). Consequently, a scattering order of three was used in all simulations. The emission of the photons was performed for 2*π* geometry and tracked by the delta-scattering method till the interaction point, where it was forced to interact by either incoherent or coherent scattering [25]. For coherent scattering, the used differential cross section is the classical Thomson differential cross section adjusted with the atomic form factor [4]. For incoherent scattering, the Klein-Nishina differential cross section was used and applied by Kahn’s method [26]. The tracking was repeated to the last scattering order. At each interaction point, a virtual photon was forced toward the detector within the permitted solid angle, see below. The weight of the photons was adjusted accordingly.

The attenuation coefficient for specific photon energies was determined from the computed tomography (CT) numbers. The CT numbers were divided into 24 intervals, ranging from − 1000 to + 1524 HU, and converted to atomic compositions using data from phantom measurements [27]. The compositions were used as input to the XCOM database in order to retrieve mass attenuation coefficients for photo absorption as well as coherent and incoherent scattering [28]. The CT numbers were also individually translated to densities, which were then combined with the mass attenuation coefficients to form attenuation coefficients.

### Collimator-detector model

The collimator-detector consisted of a 2D matrix of the crystal, with adjustable parameters for pixel size, spatial resolution and and energy resolution, and a pre-determined angular response function (ARF)-based collimator [29–31]. The ARF for different energies, polar, and azimuthal angles was determined by simulation of the photon path through the collimator-detector model. A voxelized model of the collimator was created and used for these simulations, voxel sides of 0.08 mm. The collimator size was 30 × 30 cm^2^ and contained parallel hexagonal holes. The hole size, septa, and length of the modeled medium-energy collimator for the used GE Hawkeye camera were 3.0, 1.05, and 58 mm, respectively. Within a 3 × 3-cm^2^ area in the central part of the collimator model, 1000 photons for each combination of photon energy (10–600 keV in the step of 1 keV), polar (0–1 in the step of 1/512), and azimuthal angle (0–2*π* in the step of π/256) were emitted from randomly selected positions at the collimator surface, i.e., for all combinations more than 10^11^ photons were emitted. The photon path through the collimator to the crystal was followed as described above, but the scattering was not included. The weight of each photon energy, polar, and azimuthal angle was recorded and saved as tabulated values. The tabulated values were used as look-up tables for determining the weight and interaction position in the crystal of the incoming photon. This approach is a fast method for generating the effect of septal penetration and obtaining the characteristic star effects in the image. However, the collimator and backscattering are not included in the ARF. In SARec, we included these effects by applying a Gaussian scattering kernel, which had two parameters, i.e., the weight of the signal in the voxel that was to be smeared out by the kernel and the standard deviation of the kernel. The parameters of the photon scattering kernel were determined by comparing the SARec-simulated line profiles against the gamma camera-measured line profiles in the air (see below). The best parameter settings that produced minimal divergence between the areas under the curve for the simulated and measured line profiles were used in the collimator model. In all simulations, the photon energies detectable in a 20% energy window over the 208 keV photon peak, i.e., the 208, 250, and 321 keV photons, were simulated.

### Gamma camera measurements

Line sources with ^177^Lu solutions (0.3 MBq/ml) were prepared and measured in air at three different distances (5, 10, and 15 cm) from the gamma camera collimator. The gamma camera used for generating planar and SPECT/CT images was a Millennium VG Hawkeye (General Electric Medical Systems, Milwaukee, WI, USA), equipped with a medium-energy parallel-hole collimator. A 20% energy window over the 208 keV photon peak was used. A 1024 × 1024 matrix was used for the gamma camera measurement of the line sources. The SPECT image acquisition used the same energy setting as above. The clinical SPECT images of patients, injected with 7.4 GBq ^177^Lu-DOTATATE, were collected 24 h post-injection, with a 30-s frame time duration for 120 projections. The matrix size was 128 × 128 with a pixel size of 4.42 mm and a slice thickness of 4.42 mm. The CT images were acquired using a 140-kV tube voltage, 2.5 mAs, and a rotation speed of 2.6 rpm. The matrix size was 512 × 512 with a pixel size of 0.98 mm and a slice thickness of 5 mm.

The Jaszczak image quality phantom was used to evaluate the performance of the MC OSEM reconstruction (SARec-OSEM) in comparison with standard clinical attenuated corrected OSEM reconstructions (AC-OSEM) and clinical state-of-the-art OSEM reconstructions with resolution recovery corrections (RRC-OSEM). A 256 × 256 matrix was used in this evaluation. The six spheres and the background in the Jaszczak phantom were filled with an activity concentration of 300 and 12 kBq/ml, respectively. Thereby, the activity concentration ratio between the spheres and the background was equal to 25, which is in the order of the tumor-to-normal tissue ratios observed in patients [32]. The activity recovery, i.e., the normalized signal-to-background ratio (SBR) and signal-to-noise ratio (SNR), were measured. The signal in the different sphere sizes was measured within a volume of interest (VOI) equal in size to the spheres. The mean background and the standard deviation of the background were measured in 19 VOIs, equal in size to the signal VOI, and placed in the central plane of the phantom.

## Results

**Fig. 1:**
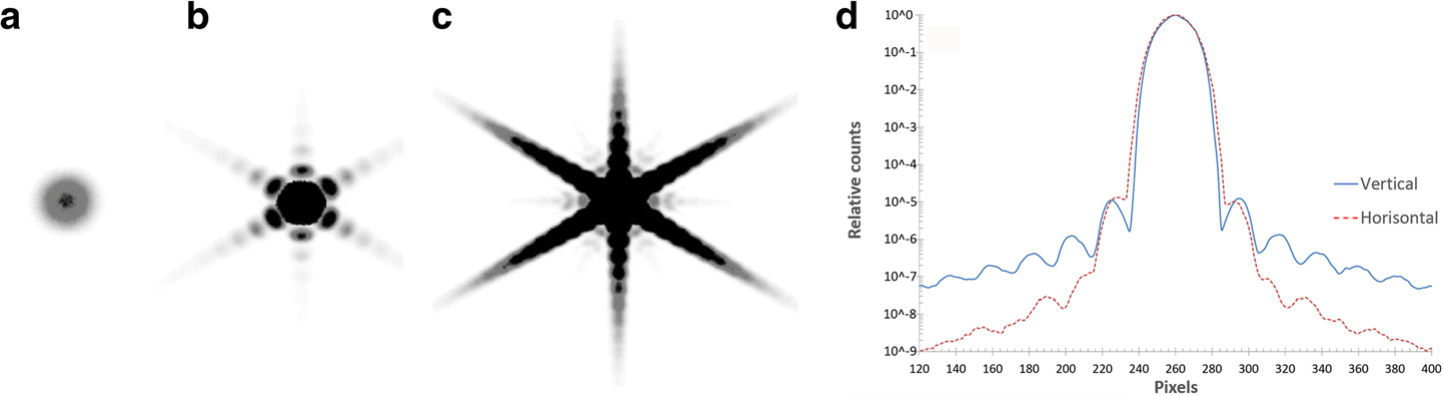
MC simulation of a gamma camera image of a ^177^Lu point source revealing a low-intensity star pattern. Window settings from non-saturated values (**a**) to pronounced saturation of the high signal values (**b**, **c**). The relative counts versus pixels for vertical and horizontal line profiles through the center of the point source (**d**)

The novel GPU-based Monte Carlo simulation code SARec was robust and stable, and its performance was 3200 million photons/s. The generation of the ARF was performed within 8 min and implemented as look-up tables in SARec. Simulation of a point source with the ARF implemented into SARec resulted in the characteristic star pattern with low intensity (Fig. 1). This low intensity justifies that the forced photon emission toward the detector could be emitted within a rather small solid angle, determined as the angle between the physical hole length and an effective hole size of 3.5 mm (compared to the physical hole size of 3.0 mm).

**Fig. 2:**
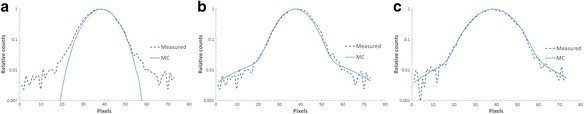
MC simulation of a gamma camera image of ^177^Lu line sources revealed similar profiles as the measured line profiles. **a** The simulated line profiles when using an ARF without scattering kernel, 5 cm from the collimator surface (observe the logarithmic scale). **b**. **c** The simulated line profiles when using an ARF with a scattering kernel, 5 and 10 cm, respectively, from the collimator surface

The SARec simulation of a gamma camera image of ^177^Lu line sources revealed similar profiles as the measured line profiles (Fig. 2). The ARF without scattering kernel agreed well with the main intensities from the line source, but could not correctly capture the photon scattering in the detector (Fig. 2b). Adding a scattering kernel with 8% of the signal and a standard deviation of 9 to the ARF also captured the photon scattering in the detector (Fig. 2b, c). The agreements between the measured and simulated line profiles, as determined by the areas under the curve at different collimator-to-line source distances, were within 3%.

**Fig. 3:**
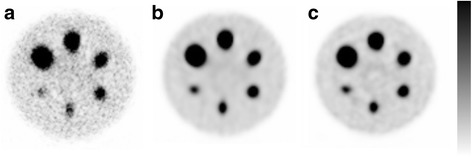
Reconstructed SPECT images of the Jaszczak phantom filled with ^177^Lu activity in the spheres and the background. The activity concentration ratio between the spheres and background was equal to 25. **a** AC-OSEM. **b** RRC-OSEM. **c** SARec OSEM images

SARec was implemented in an OSEM reconstruction of ^177^Lu SPECT/CT images (Fig. 3). The stability of the reconstruction was slightly improved with an increased number of emitted photons/voxel, and the reconstruction time was linearly dependent on that number. The required number of emitted photons/voxel to obtain a stable reconstruction was estimated by the coefficient of variation (CV) for the different numbers in a 22-mm background sphere in the phantom, using ten subsets and six iterations. The CV was reduced until about 200 photons/voxel, and a minor improvement in CV was obtained thereafter. In continuing studies, simulations were performed with 200 photons/voxel in a 128^3^ voxel matrix for clinical studies and in a 256^3^ voxel matrix for phantom studies. With this number of emitted photons/voxel, the SARec OSEM reconstruction with ten subsets was performed within 20 s/iteration for the clinical studies and eight times longer for the phantom measurements.

**Fig. 4:**
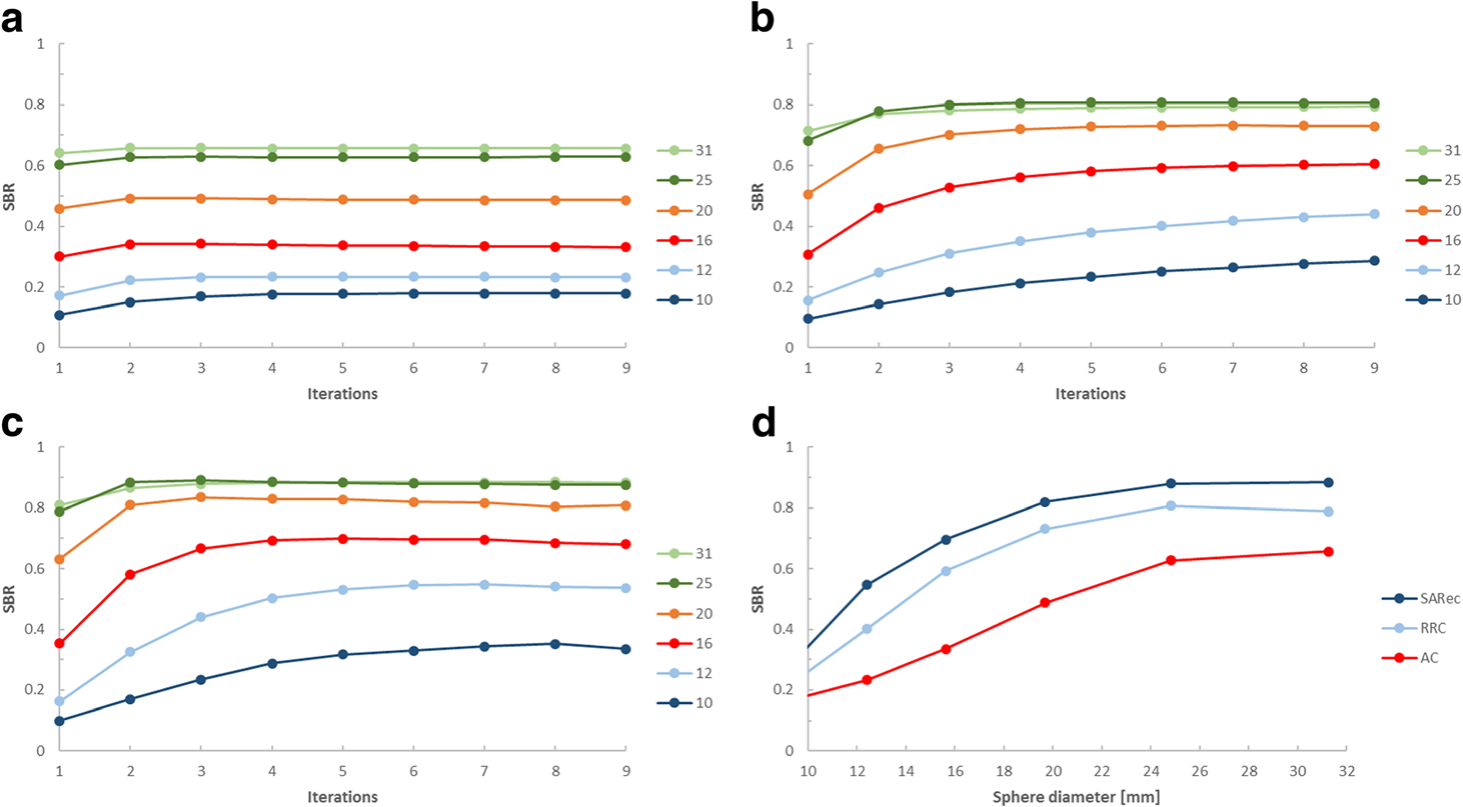
The activity recovery (or normalized signal-to-background ratio (SBR)) for the different SPECT reconstructions versus the number of iterations of the ^177^Lu-filled Jaszczak phantom. **a** AC-OSEM. **b** RRC-OSEM. **c** SARec OSEM. The curves show the SBR versus the number of iterations for the six spheres with diameters 10, 12, 16, 20, 25, and 31 mm, respectively. In panel **d**, the SBR versus sphere diameter for six iterations are shown for the AC-OSEM (AC), RRC-OSEM (RRC), and SARec OSEM (SARec) reconstructions

In the phantom measurements, the number of iterations for obtaining recovery convergence was dependent on the sphere sizes in the phantom, where the larger spheres converged faster than the smaller ones (Fig. 4). There was also a difference in convergence rate between the reconstruction methods. For AC-OSEM and SARec OSEM, the recovery convergence, for all spheres, was reached after about six iterations. However, for the smallest sphere, a small increase in recovery could still be noted even after six iterations. With RRC-OSEM and six iterations, four of the six spheres reached the recovery convergence; all spheres reached recovery convergence after nine iterations. For the final analysis, six iterations and ten subsets were used; with this number of iterations and subsets, the SARec OSEM reconstruction time of the clinical images was around 3 min.

The activity recovery for the spheres in the phantom was improved with the SARec OSEM reconstruction, e.g., for ^177^Lu; the activity recovery for the largest sphere and six iterations was 88%, compared to 79 and 66% for RRC-OSEM and AC-OSEM, respectively (Fig. 4d). With SARec OSEM, the spheres appeared to be more spherical, compared to RRC-OSEM (Fig. 3). For SARec, the background appeared to be less uniform than RRC-OSEM, and the mean signal-to-noise ratio (SNR) for all spheres was 12% lower than for RRC-OSEM. The corresponding mean SNR for AC-OSEM was 25% lower than RRC-OSEM.

**Fig. 5:**
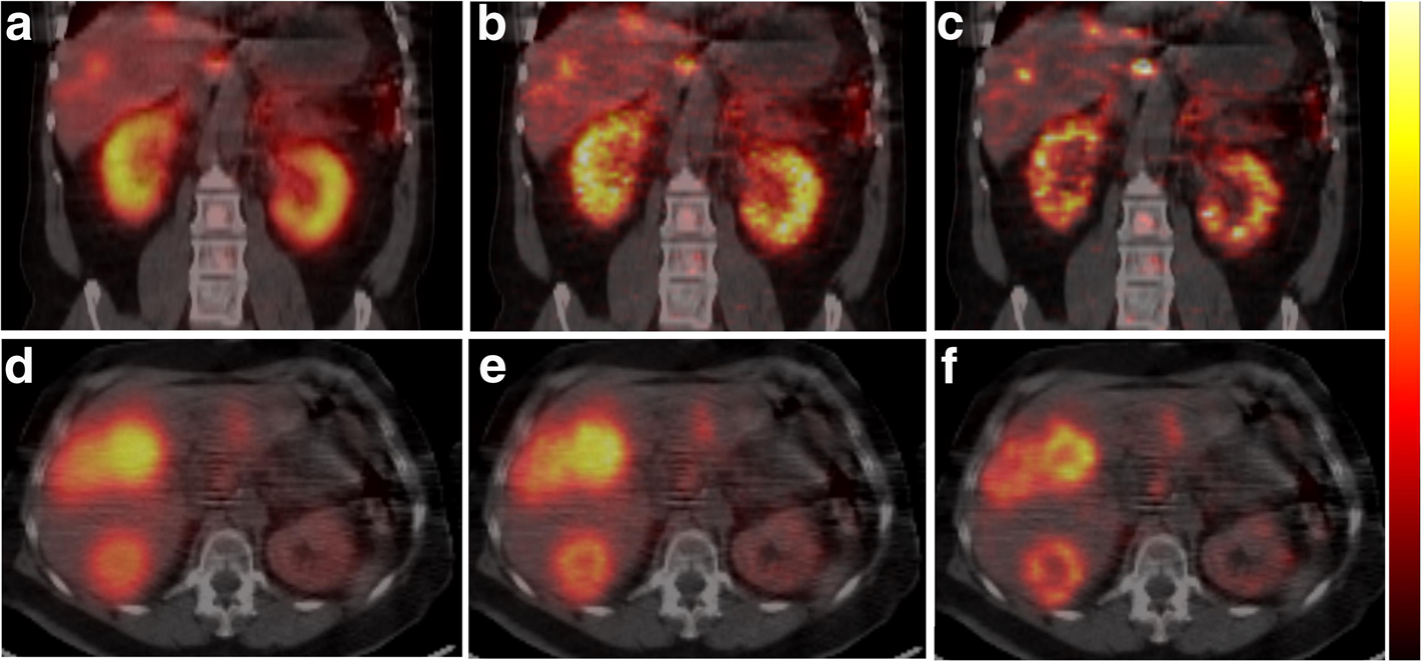
SPECT reconstructions of the ^177^Lu distribution in two patients treated with ^177^Lu-DOTATATE. Panels **a**, **d** show the post-filtered AC-OSEM reconstructed SPECT images that are used in the clinical protocol, while panels **b**, **e** show SPECT images reconstructed with unfiltered AC-OSEM, and panels **c**, **f** show SPECT images reconstructed with SARec-OSEM. In the Monte Carlo-based reconstruction with SARec, an increased number of tumors and an improved visualization of the heterogeneous activity distribution in the kidney were observed (**c**), which become more evident when a post-filter is applied (data not shown). Also, in the tumor tissue, the improved heterogeneous activity distributions were more easily observed with SARec OSEM compared with AC-OSEM, an example is the marked uptake differences between the tumor border and tumor center (**f** versus **d** and **e**)

**Fig. 6:**
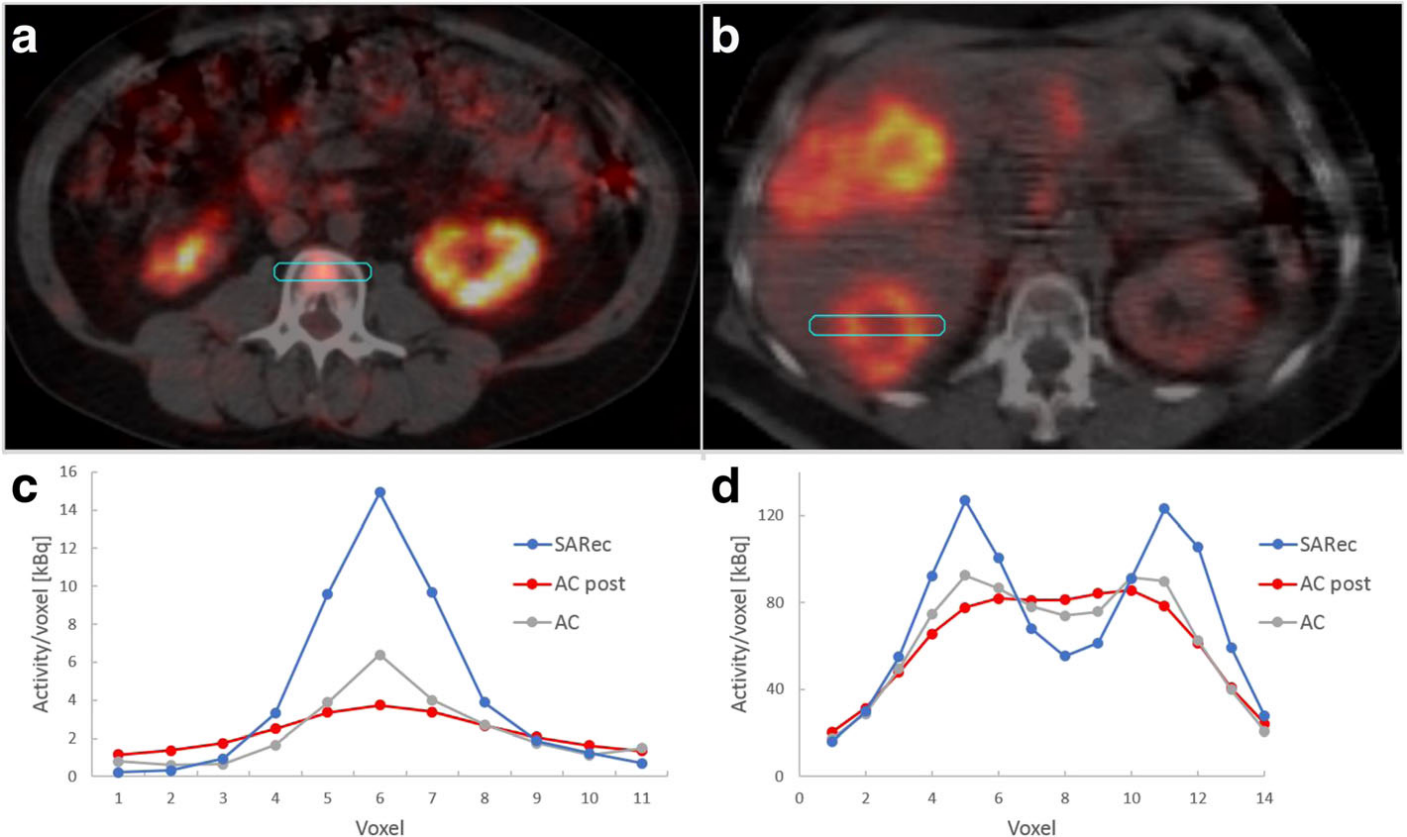
Transverse sections of SARec reconstructed images with line profiles through a bone marrow uptake (**a**, **c**) and a liver tumor with non-uniform uptake (**b**, **d**). The line profiles show the uptake profiles for SARec (SARec) and AC-OSEM (AC) post-filtered AC-OSEM (AC post) reconstructed images

Figure 5 provides illustrative examples of the improved image quality with SARec OSEM (c, f) compared with AC-OSEM (b, e) and post-filtered (Butterworth; power factor 2 and 0.048 cycles/mm) AC-OSEM (a, d), which are used in many clinical protocols for patients undergoing treatment with ^177^Lu-DOTATATE. The visualization of multiple liver tumors, the non-uniform activity distribution in the kidney cortex, and the non-uniform activity distribution in the tumors (D-F) were improved using SARec OSEM compared with the visualization using AC-OSEM. In Fig. 6, line profiles (c, d) through the bone marrow uptake observed in Fig. 5a–c and the dorsally located tumor in Fig. 5c, d are demonstrated, together with the SARec reconstructed transversal sections through the tumors (a, b). The line profiles demonstrated the improvement in determining non-uniform activity distribution in SARec reconstructed images.

## Discussion

This study shows that a new GPU-based Monte Carlo simulation algorithm for producing forward projections in the SPECT reconstruction can generate high-quality clinical images within 3 min, with a clearly improved recovery compared with attenuated corrected OSEM reconstruction and with similar recovery as the clinically used state-of-the-art reconstruction with resolution recovery corrections.

The concept of using a Monte Carlo simulation to produce the forward projection has been highlighted by several authors and implemented in a few cases [7–9]. In previously developed MC codes, several variance reduction techniques were implemented to speed up the reconstruction times, but since these codes were written for the CPU, there was only limited ability to parallelize the codes, and thus, the required reconstruction times remained unacceptably long for clinical use. However, the execution speed in a CPU-based state-of-the-art workstation can be high, e.g., with eight CPU processors having ten cores each and running in hyper-threading, the performance will be about nine·10^12^ floating-point operations per second (FLOPS). Nevertheless, the execution speed for a MC code written for a GPU-based workstation will be higher, about 44·10^12^ FLOPS with four GPUs. In addition, the cost of a state-of-the-art CPU-based workstation is about 10 times higher than for a corresponding GPU-based workstation, resulting in a performance/cost ratio that is about 50 times higher for a GPU-based workstation compared to a state-of-the-art CPU-based workstation.

Recently, Garcia et al. [10] introduced GPU Geant4-based Monte Carlo simulations, and they demonstrated a speed-up factor of around 70 compared to CPU-based programming in the SPECT reconstruction of an anthropomorphic phantom. This improvement in simulation time was due to the use of parallel programming for the millions of threads in the GPU. However, even with this positive result for GPU programming in MC-based SPECT reconstruction, the simulation times were still too long. Effective variance reduction techniques are required to further reduce the simulation times in MC-based SPECT reconstructions. In the SIMIND code, which is specifically designed for effective simulation of gamma camera images, several effective variance reduction techniques for photon transport have been implemented, and the code has been verified in several studies [1].

In our GPU code, we used variance reduction techniques for photon transport as described for SIMIND and used a modified version of the analytical function for the geometrical response of the collimator, as first proposed by Metz et al. [29, 30] and extended to the simulated ARF as proposed by Song et al. [33]. In the original collimator model, the solid angle of the emitted photon is limited to the maximum angle determined by the geometry of the collimator hole. This enabled a speedup of the simulation times since the photons can be forced into a small solid angle. However, such model is not able to account for septal penetration and scattering in the collimator and detector, or for the interaction in the crystal. These effects are complex and time-consuming to simulate, and different approaches have been used in their approximation [33, 34]. In our approach, we simulated the penetration effects and adjusted the collimator scattering with a Gaussian kernel that was determined from measured line sources at different collimator-to-source distances. The agreement between the measured and simulated line sources was rather good, i.e., less than 3% divergence.

The advantage with this fast, but approximated, collimator-detector response model is that the main attenuation differences for the photon transport within the solid angle are taken into account. In contrast, the commonly used forced convolved method is based on the photons being forced into a perpendicular angle toward the crystal, and a pre-simulated distance energy-dependent Gaussian kernel is applied which speeds up the reconstruction time but will not take into account the variable attenuation within the solid detection angle [35]. A direct comparison between these two approaches has not been performed in this study but will be the goal for later analysis of the differences in reconstruction times and image quality, with focus on activity quantification aspects for radionuclide therapy.

Our collimator-detector response model includes both attenuation correction and variable polar and azimuthal angle septal penetrations—which are important for high energetic photons such as the 364 keV photons emitted from ^131^I. However, accurate ARF requires a substantial simulation of all parameter values, which are not always easily implemented in the simulations, e.g., the commonly used energy resolution model might not be useful for high-energy photons and the complex structure of the backscattering components makes the simulation challenging [36, 37]. Despite the complexity of simulating ARF correctly, the polar and azimuthal angular-dependent ARF are important for high energetic photons, and it would be valuable to further study their impact on simulation times and image quality, which can be performed effectively by using the fast simulation performance of SARec.

The presented MC-based SPECT reconstruction method showed superior image quality compared to the attenuated corrected OSEM reconstruction. The method seemed to be able to correct the scattering and improve the resolution. Consequently, with SARec, the improvement of tumor visualization and non-uniform activity distribution in tumors and organs like the kidney are obtained. Such improvement in activity distribution estimates is important in the continuing development of dosimetric modeling of individual response estimates, e.g., by using the EUBED in radionuclide therapy. Furthermore, the improved quantification of non-uniform activity distribution also addresses the drawback of using segmentation methods based on the assumption that the activity is uniformly distributed, e.g., the 42% iso-contour method [15]. These methods will only capture the fraction of tissue with the highest uptake and exclude the low uptake areas. Similar improvement in image resolution, as that obtained with SARec, was also obtained with resolution recovery corrected OSEM reconstructions. However, since RRC algorithms are based on pre-simulated energy and distance-dependent kernels of the image blurring, where the forward projection is convolved with the kernels slice by slice in the 3D matrix, Gibbs artifacts are generated and will influence quantitative estimates of the activity distribution [20, 21]. Furthermore, in this study, we also observed that the spheres seem to become elliptical when using RRC, indicating that the distance dependence is not fully described by the approximated kernels. Therefore, fast and accurate MC-based SPECT reconstructions are important tools in the future development of accurate dosimetry and response modeling for radionuclide therapy.

The SARec code was run on GPUs with single precision, and this was shown to provide similar image quality results as those obtained with double precision used in GPU-based SPECT reconstruction with GEANT4 [10]. In SARec, we used four GPUs since this provided a trade-off between effectiveness and cost. With more GPUs, the speed would be proportionally improved, but a more advanced arrangement would be needed with higher relative cost of the computer components. The use of four or fewer GPUs requires only standard components for a personal desktop. Furthermore, the speed of GPUs is still rapidly increasing, and the next version of Nvidia GPUs should have twice the capacity as the GPUs used in this study. We, therefore, conclude that four GPUs should be enough for the future expansion of MC-based SPECT reconstructions into daily clinical use.

## Conclusions

The use of established variance reduction techniques for photon transport and effective collimator-response modeling in a GPU-based Monte Carlo code for SPECT reconstruction can produce substantially improved images within a few minutes, which highlights its potential for clinical use, even in routine applications.

## Abbreviations

AC: Attenuated corrected
ARF: Angular response function
CPU: Central processing unit
CT: Computed tomography (CT)
CUDA: Compute Unified Device Architecture
CV: Coefficient of variation
EUD: Equivalent uniform dose
FLOPS: Floating-point operations per second
GPU: Graphics processing units
MC: Monte Carlo
OSEM: Ordered subset expectation maximum
RRC: Resolution recovery corrections
SARec: Sahlgrenska Academy reconstruction code
SBR: Signal-to-background ratio
SNR: Signal-to-noise ratio
SPECT: Single-photon emission tomography
TEW: Triple energy window
VOI: Volume of interest
